# Clinical exploration of flexible ureteroscopy combined with holmium laser and intraoperative ultrasound localization for the treatment of stones in non-communicating calyceal diverticula: a single case report

**DOI:** 10.3389/fsurg.2026.1799215

**Published:** 2026-03-30

**Authors:** Qiang Wang, Yiwei Xu, Lang Cheng, Xiaopeng Chen, Houbao Huang, Ping Ao

**Affiliations:** Department of Urology, The First Affiliated Hospital of Wannan Medical College, Wuhu, China

**Keywords:** anatomical anomaly of the ureteral orifice, flexible ureteroscopy, holmium laser, intraoperative ultrasound, non-communicating renal calyceal diverticulum stones

## Abstract

**Objective:**

To investigate the value of intraoperative multimodal localization strategies in managing non-communicating calyceal diverticulum stones without preoperative definitive diagnosis in a single clinical case.

**Methods:**

A 48-year-old female with a history of open renal stone surgery and cesarean section was enrolled in this single case study. Preoperative CT revealed a calcified lesion (1.5 × 1.2 cm, CT value 1,235 HU) at the right upper renal pole but failed to identify the diverticular structure. Intraoperative exploration identified a ureteral orifice ectopically located on the right posterior bladder wall (23 mm deviation from midline). An innovative “three-step localization protocol” was implemented: 1. CT-anatomical landmark spatial co-registration, 2. Guidewire advancement with tactile feedback validation, 3. Real-time ultrasonographic confirmation of guidewire tip positioning. A 10/12Fr ureteral access sheath was pre-placed in the right ureter to establish a stable operative channel, facilitate irrigation and suction, and reduce ureteral mucosal injury during the surgical procedure. Diverticular neck incision was performed using holmium laser (200 μm fiber, 0.8J/20 Hz) for channel creation, followed by stone fragmentation under negative-pressure suction system guidance.

**Results:**

The procedure was completed in 90 min with negligible blood loss (<10 mL). Intraoperative ultrasonography confirmed complete stone clearance. No postoperative complications or recurrence was observed during follow-up.

**Conclusion:**

Multimodal localization technique achieved effective and precise surgical navigation for the non-communicating calyceal diverticulum in this single case. This approach combines the advantages of natural orifice preservation with controlled infection risk and minimized hemorrhagic complications, and may provide a valuable clinical and technical reference for cases with atypical anatomy and inconclusive preoperative imaging.

## Introduction

Calyceal diverticulum, a congenital or acquired cystic lesion of the renal parenchyma, is characterized by a narrow-necked connection to the collecting system and a lining of nonsecretory transitional epithelium (1). Epidemiological studies indicate that 9.5–50% of cases develop secondary calculi due to urinary stasis, with completely obstructed (non-communicating) diverticular stones accounting for 1.2–1.8% of symptomatic nephrolithiasis cases (2). Stone composition analyses reveal that mixed calcium phosphate/oxalate stones predominate (72–85%), with pathogenesis involving both urine supersaturation secondary to stasis and IL-6/TNF-α-mediated pro-mineralization inflammatory pathways (3).

Clinically, approximately 60% of patients present with flank pain or recurrent urinary tract infections, while 30% are incidentally detected on imaging. Only 10% manifest gross hematuria or pyuria (4). Although CT urography (CTU) remains the gold standard for diagnosis (5), its sensitivity for non-communicating diverticula is markedly reduced. Existing literature has reported that 40% of cases exhibit “pseudo-parenchymal renal calculi” on CT imaging due to fibrotic neck closure or beam-hardening artifacts from calcified stones, leading to preoperative mislocalization in 19–26% of cases (6).

Percutaneous nephrolithotomy (PCNL) persists as the first-line intervention despite inherent risks. The requirement for percutaneous tract establishment increases parenchymal trauma and perioperative morbidity compared to flexible ureteroscopy, with meta-analyses demonstrating relatively higher rates of bleeding (14.8% vs. 4.1%) and infectious complications (10.3% vs. 3.6%) (7, 8).

## Case presentation

**Figure 1:**
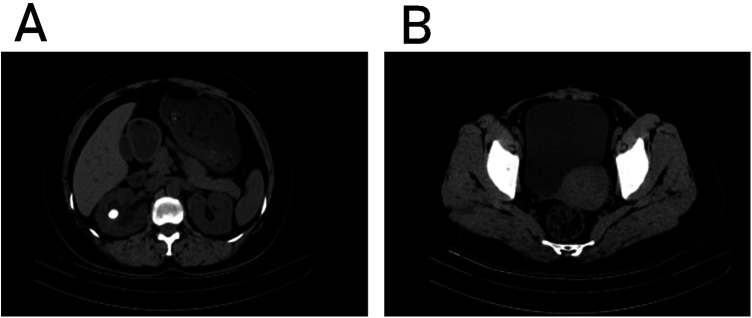
**(A)** Calyceal diverticulum stone location. **(B)** The bladder is squeezed by the uterus.

A 48-year-old female presented with a 4-day history of right flank pain, without urinary frequency, urgency, dysuria, nausea/vomiting, or fever. Surgical history included open right nephrolithotomy 37 years prior and cesarean section 19 years prior. Physical examination revealed right costovertebral angle tenderness. Non-contrast CT demonstrated a hyperdense lesion (CT value 1,235 HU) in the mid-upper calyx of the right kidney (Figure 1A) and dense adhesions between the anterior uterine wall and posterior bladder wall, with rightward bladder deviation (Figure 1B).

### Surgical methods

**Figure 2:**
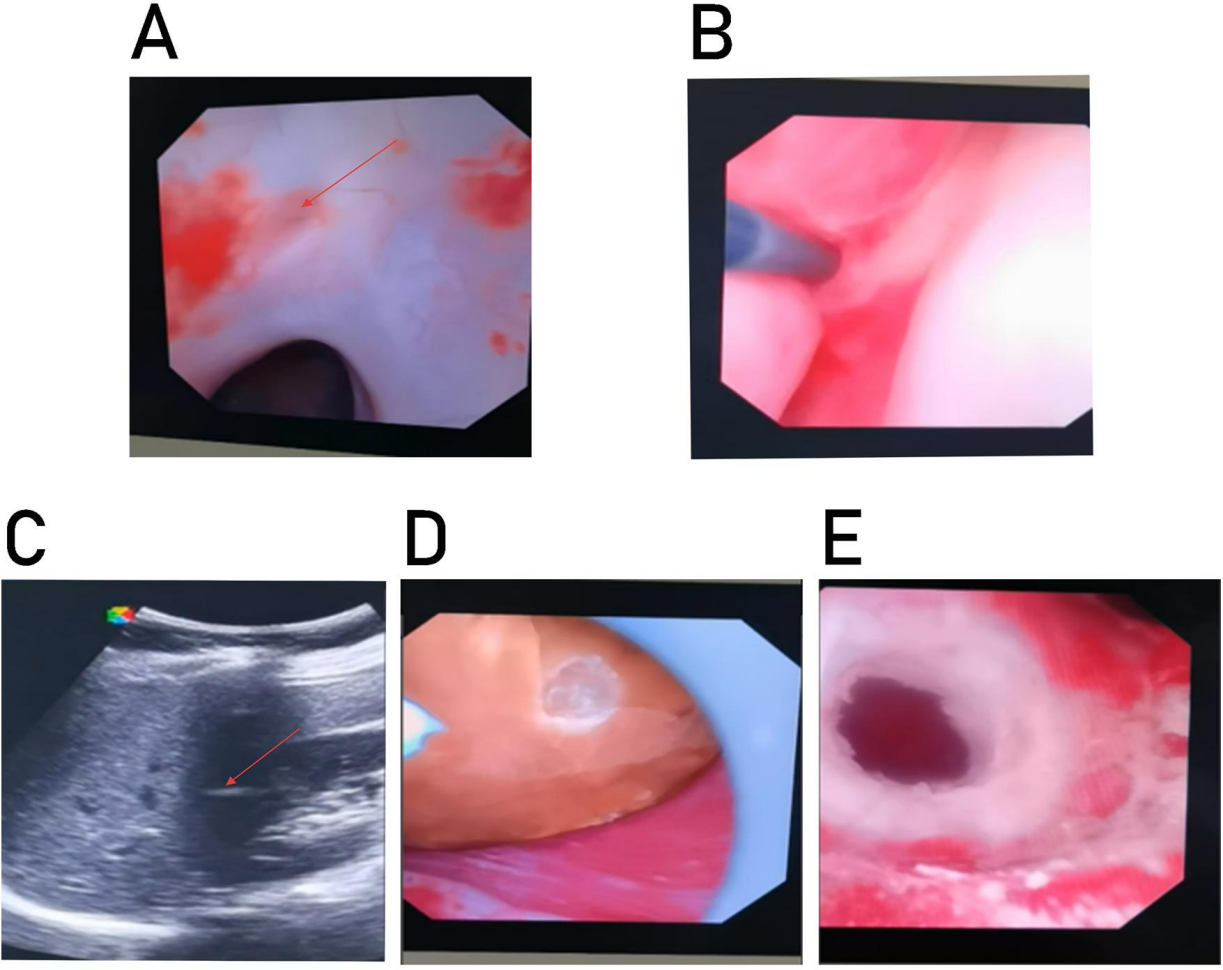
**(A)** Suspected non-communicating diverticulum orifice. **(B)** The resistance at the tip of the guidewire decreases sharply into the cavity. **(C)** Ultrasound positioning. **(D)** Yellow-brown oval stones were found. **(E)** Circumferential incision of the diverticulum opening.

Under general anesthesia, the patient was placed in lithotomy position. After standard disinfection and draping, a rigid ureteroscope was introduced into the bladder, revealing no calculi or neoplasms. Anatomical distortion secondary to prior cesarean section resulted in left ureteral orifice displacement to the right bladder wall (23 mm from midline), complicating ureteral identification. Systematic calyceal exploration via bilateral ureters failed to locate the calculus. A 7.5Fr flexible ureteroscope (Hunan Lingkang Medical Technology Co., Ltd.) was adopted for subsequent precise intrarenal exploration, and a 10/12Fr ureteral access sheath (Suzhou Huamei Medical Device Co., Ltd.) was placed in the right ureter due to ureteral stricture to maintain a clear operative channel and optimize the efficiency of irrigation and suction throughout the procedure. We subsequently implemented a three-step anatomical-electronic localization protocol: 1. Anatomical Correlation: Identified a suspected non-communicating diverticular orifice at the CT-mapped calculus projection zone (Figure 2A). 2. Tactile-Guided Access: A fibrotic membrane-covered depression was probed with a zebra guidewire, confirming entry into a cavity by sudden loss of resistance (Figure 2B). 3. Sonographic Verification: Intraoperative ultrasound confirmed guidewire tip-coincidence with the calculus (Figure 2C). Intraoperatively, a normal saline perfusion pump system was used with a preset pressure of 40 mmHg and a flow rate of 100 mL/min to maintain a clear surgical field of vision and regulate the renal pelvic pressure. Intraoperative observation was focused on the filling status of the renal pelvis and calyces, which were consistently kept in a semi-filled state during the procedure. Meanwhile, unobstructed drainage of the negative-pressure suction sheath was ensured throughout the operation. However, real-time monitoring of the renal pelvic pressure and intra-operative temperature was not performed due to the lack of relevant monitoring equipment. Retrograde pyelography was not performed to reduce contrast medium exposure and shorten the operative time; instead, the combination of guidewire tactile feedback and real-time high-resolution intraoperative ultrasound was used to repeatedly confirm the guidewire tip position, thereby avoiding the risks of renal parenchymal perforation and false passage formation. The diverticular neck was radially incised using holmium laser (0.8 J, 20 Hz) to achieve an approximately 10Fr passage (Figure 2D). The holmium laser fiber (200 μm) was manipulated in a gentle radial pattern during the incision to minimize thermal injury to the surrounding renal parenchymal tissues. A 15 mm ovoid yellowish-brown calculus (Figure 2E) was fragmented via central vaporization-drill technique to sub-2 mm fragments under continuous low-pressure suction. Total operative time was 90 min (laser activation: 16 min) with <10 mL blood loss.

### Postoperative course

The patient demonstrated resolution of flank pain and was discharged on postoperative day 2. Follow-up imaging at 4 weeks confirmed complete stone clearance without diverticular recurrence.

## Discussion

### Diagnostic challenges of non-communicating calyceal diverticulum stones

The core diagnostic challenge in this case lies in the difficulty of preoperative identification of non-communicating calyceal diverticular stones. Although CT non-contrast and enhanced scans remain the gold standard for imaging, they failed to delineate the diverticular anatomy in this patient, likely due to inflammatory fibrosis-induced occlusion of the narrow channel in non-communicating diverticula. Notably, 15–30% of diverticular stone cases lack classic CT features such as the “papillary depression sign” or “narrow neck sign” (9). The patient's history of prior open surgery and altered pelvic anatomy further obscured imaging findings. Post-cesarean uterine leftward deviation may compress the bladder, leading to ureteral orifice displacement, underscoring the importance of supplementary diagnostics (e.g., 3D urographic reconstruction or retrograde pyelography) in patients with complex surgical histories. These anatomical variations compounded the complexity of endourological access.

### Intraoperative localization breakthrough via tri-modal technique

A three-step localization strategy was applied in this case to address the intraoperative identification challenge of non-communicating diverticula: 1) Anatomical Landmark-guided Spatial Mapping: Merging real-time operative field anatomy with preoperative imaging projections, overcoming the traditional reliance on identifying narrow-neck structures. 2) Dynamic Guidewire Probing with Tactile Feedback: Utilizing sudden “loss of resistance” upon penetrating fibrous septa for objective confirmation of hidden cavities. 3) Ultrasound-Validation of Guidewire Tracking: Real-time intraoperative ultrasound synchronized with guidewire tip movement, achieving sub-2 mm spatial accuracy. This multimodal localization approach provides a practical and precise intraoperative navigation method for minimally invasive urological surgery in similar complex cases with anatomical distortion.

### Evolution of minimally invasive pathways

While percutaneous nephrolithotomy (PCNL) remains standard for symptomatic diverticular stones, a natural orifice-based endoscopic approach achieved favorable clinical outcomes in this single case, with the following advantages: 1) Ultra-micro access (<10Fr) via flexible ureteroscopy with holmium laser lithotripsy, minimizing puncture-related hemorrhage/infection risks; 2) Low-pressure intracavitary irrigation via negative-pressure suction combined with ureteral access sheath drainage, reducing postoperative urine extravasation; 3) Preservation of renal parenchymal integrity, avoiding potential long-term renal functional compromise associated with PCNL. Intraoperative blood loss in this case was <10 mL, which is consistent with the reported lower bleeding rate of flexible ureteroscopy compared to PCNL in existing literature.

It is important to emphasize that each surgical approach has its own unique risk profile. PCNL features a clear operative channel established under radiological guidance, with mature and standardized surgical procedures; while the flexible ureteroscopy approach in this case relies on the combination of ultrasound and tactile feedback for navigation, with a theoretical risk of parenchymal perforation or false passage formation in fibrotic and previously operated kidneys. No such complications occurred in this case due to strict intraoperative navigation standards, repeated confirmation of guidewire tip position and the protective effect of the ureteral access sheath on the ureteral and renal parenchymal tissues (10–14).

### Clinical implications

Existing published studies have reported that the success rates of flexible ureteroscopy and PCNL for diverticular stones are comparable (84% vs. 89%) in recent 5 years (15, 16). This case provides two important clinical references for the management of non-communicating calyceal diverticulum stones with preoperative imaging limitations and anatomical distortion: 1) Effective localization and successful minimally invasive treatment can be achieved through the three-step localization strategy even when preoperative imaging fails to clearly identify the diverticular structure; 2) A standardized operational protocol combining holmium laser lithotripsy, intraoperative ultrasound localization, guidewire tactile feedback and ureteral access sheath application is feasible and safe for such complex cases.

### Limitations of this case

This study is a single case report, and thus the clinical outcomes and technical feasibility of the three-step localization strategy cannot be generalized to all patients with non-communicating calyceal diverticulum stones. In addition, the operation was performed in a tertiary hospital with experienced urological surgeons and high-precision intraoperative ultrasound equipment, which may limit the reproducibility of this technique in primary medical institutions with relatively limited medical resources and technical experience. Further clinical studies with larger sample sizes (e.g., case series or retrospective cohort studies) are needed to verify the safety and efficacy of this approach in a broader patient population.

## Conclusion

This single case report demonstrates that the combination of flexible ureteroscopy, holmium laser lithotripsy, intraoperative ultrasound localization and ureteral access sheath application is a feasible and effective minimally invasive treatment option for non-communicating calyceal diverticulum stones with atypical anatomy and inconclusive preoperative CT imaging. The three-step localization strategy (CT-anatomical landmark correlation, guidewire tactile feedback, real-time ultrasound confirmation) achieved precise intraoperative navigation with a small positioning error in this case, and the integrated fragmentation-aspiration protocol combined with ureteral access sheath drainage effectively maintained a safe renal pelvic pressure and minimized intraoperative blood loss (<10 mL). Prechannel-free diverticular neck plasty with holmium laser also avoided additional parenchymal trauma in this patient.

This approach provides a valuable clinical and technical reference for the management of complex non-communicating calyceal diverticulum stones, especially those with anatomical distortion due to prior surgery. However, due to the inherent limitations of the single case design, the safety and efficacy of this technique need to be further verified by more clinical cases and follow-up studies. Future enhancements through virtual imaging navigation and tactile-sensing guidewire technology could further improve the localization efficacy for fibrotic-occluded diverticula in clinical practice. This surgical approach may serve as a supplementary minimally invasive option for the clinical management of complex non-communicating calyceal diverticulum stones.

## Data Availability

The original contributions presented in the study are included in the article/Supplementary Material, further inquiries can be directed to the corresponding authors.
